# True Croup; or Pseudo-Membranous Laryngitis

**Published:** 1879-01

**Authors:** J. A. Goldsberry

**Affiliations:** Annapolis, Ind.


### ARTICLE III.

TRUE CROUP ; OR, PSEUDO-MEMBRANOUS LARYNGITIS. By J. A. GOLDSBERRY, ANNAPOLIS, IND. Read before the Parke County, Ind. Medical Society.

True croup, sometimes called diphtheritic, exudative, fibrinous, is an inflammation of the mucous membrane of the larynx, with an exudation upon its surface of lymph or pseudo-membrane. It is a disease which may be said to be almost peculiar to childhood, as it rarely occurs in those under two or beyond seven years of age ; no age, however, it seems, is absolutely exempt from it. It is confined to no latitude or country, but is endemic throughout the United States and all over the civilized world.

The causes which contribute to the occurrence of this fatal malady, are, I think, in many instances, obscure. The child of

|| Smith, Lectures on Obstetrics, Gardner, New York, 1858.

splendid *physique*, well fed and well clothed, living in the rural districts and surrounded by influences such as conduce to the highest degree of comfort and health, and the half-starved sickly offspring of the poor of our filthy and crowded cities, are alike subject to its attack. Some authorities are of the opinion that there exists in some children a predisposition to the disease, and that that predisposition may be inherited, which I think quite probable.

Boys are more frequently affected than girls. Unquestionably the most common exciting cause is exposure to cold; naturally then, we would expect it to occur to a greater extent in some localities than others.

A climate characterized by great and frequent fluctuations in temperature, with a cold and humid atmosphere, is productive of the disease; hence its frequency in this latitude. It is more common in the spring and winter months than in other seasons of the year. There are now and then instances in which the cause may be traumatic, as in the inhalation of scalding steam, or the swallowing of acrid and corrosive liquids. It occurs usually as a primary disease but not unfrequently it is secondary. In its secondary form it may come up in the declining stage of measles and small-pox; it may complicate diphtheria and scarlatina.

As this is, primarily at least, an acute inflammation of the larnyx, the parts affected present the usual anatomical morbid phenomena. The nature of the lesions is precisely such as results from inflammation of mucous surfaces in other parts of the body. The mucous membrane is hyperæmic, greatly thickened and usually loaded with mucus and pus. In mild cases a small surface only of the mucous membrane may be affected. In severe cases the inflammatory action extends *beneath* this membrane, involving the contiguous tissue, with the usual concomitants, serous infiltration and œdema; and from this latter condition arises one of the chief dangers. Many cases succumb to this disease, not so much by reason of the exudation as from the œdematous condition of the submucous structure. In cases of a severe grade the inflammation extends into the trachea and not unfrequently involves the bronchial tubes. The fauces may likewise become affected. But the most important anatomical element in the disease, which gives to it its distinguishing characteristics, and which adds so much to its danger, is the formation of pseudo-membrane. Of course there can be no true croup without the presence of this exudation; divest it of this feature and you rob it of its principal terror. The extent of the false membrane is co-extensive with the inflammation. Wherever the mucous membrane is undergoing inflammatory action, whether it be in the larynx, pharynx, trachea or bronchial tubes, there we find, more or less, the fibrinous deposit. There is a difference of opinion among medical men as to the nature of these membranes. While there are some eminent pathologists who insist that they are composed of fibrin, there are others, no less distinguished, who claim that there is no fibrin in them, but that they are made up of "epithelial cells, which, undergoing some form of degeneration as they are pushed forward from the mucous surface, enlarge, and appear under the microscope, as irregular blocks interlacing with each other." But whatever may be their nature, whether fibrin or something else, all must agree that their presence in the air passages is fraught with the most imminent danger. Usually, in from five to six days, if the child survives so long, the false membrane begins to soften and break down from decomposition, and may be expelled in the act of vomiting or coughing. In fatal cases that have been examined after death, the lungs were invariably found to be more or less congested; portions of them, here and there, were collapsed, while not a few of the lobules were completely hepatized. The right cavities of the heart were also distended with blood, which from its dark color, was evidently unduly impregnated with carbon.

In the vast majority of cases this disease commences very much like a common cold or coryza, for which, unfortunately, it is often mistaken. There is more or less febrile excitement, cough, constipated bowels, impaired appetite, thirst, and the child is fretful and irritable. Very soon certain symptomatic features declare themselves which are highly characteristic of the disease.

The cough, which is always present from the very beginning, becomes hoarse, dry and ringing; some describe it as barking, suppressed, stridulous. While as just observed it is one of the first symptoms, in intensity it varies greatly in different cases. I have treated cases in which, from first to last, there was comparatively little cough. Hoarseness and huskiness of the voice, like the cough, is generally one of the first observable symptoms; it persists throughout the disease, and in fatal cases, towards the close of life, the voice is entirely lost. There is usually more or less expectoration. In some cases it is abundant, in others scanty, depending upon the extent of surface involved in the inflammation. A characteristic feature of the sputum is its extreme toughness and tenacity, so much so, that it is often expelled with the greatest difficulty.

In the advanced stage of the disease, in favorable cases, the expectoration not unfrequently mixed with strips and patches of pseudo-membrane, becomes quite profuse and loses in a measure its former tough, tenacious element. The respiration is always accelerated, not to the extent however that we would expect; certainly not so much so as in other affections of the respiratory apparatus far less serious.

The pulse in the beginning varies in frequency from 120 to 130 beats per minute; in many cases is full and strong, while in others it is rapid and feeble.

As the disease progresses the gravity of the symptoms becomes marked and unmistakable. In consequence of the increasing laryngeal, and, it may be, tracheal obstruction, the breathing is labored and difficult. Along with the mechanical impediment to respiration there seems to be a neuropathic or nervous factor, which affecting the larnyx, produces spasmodic contraction of its muscles, and adds no little to the distressing paroxysms of dyspnœa so common in the latter stage of this disease.

In fatal cases death usually occurs in five or six days; in those of unusual severity, however, life may terminate as carly as the second or third day.

The mode of death is of course, at least in the majority of instances, by asphyxia; a few die seemingly from pure exhaustion. In some, death is peaceful and quiet; in others life goes out during the throes of a violent convulsion. Unless a comatose condition should supervene, which rarely occurs, the intellect generally is clear to the last.

As this is a disease of almost unparalled fatality it is of the utmost importance that a correct diagnosis be made in the very beginning, as everything, so far as a successful result is concerned, depends upon *early* treatment. The affection for which, perhaps, more than any other it is most frequently mistaken is spasmodic laryngitis, sometimes called catarrhal or false croup. While in some particulars the two diseases simulate each other very much, it will be found that there are diagnostic symptoms in one which are not present in the other, and, which carefully observed, will almost invariably lead to a correct differential diagnosis.

The one is rather gradual in its inception, with symptoms not unlike those in a common cold, but steadily increasing in severity; the other commences, as a rule, in the night, abruptly, generally without any premonitory symptoms, followed very soon by a rapid improvement. In the one the cough, generally very troublesome, is eminently *croupal*, that is, *dry and hoarse;* a cough which it is difficult to describe in words, but which once heard is not likely to be forgotten. In the other the cough is not nearly so frequent and is wanting in that singularly dry, harsh, ringing sound so characteristic of the former. In the one, on inspection of the throat, in almost every case you will discover low down in the fauces the pseudo-membrane, also fragments of this, sooner or later, may be seen in the sputum.

Croup is sometimes confounded with internal convulsions or laryngismus stridulus. This mistake ought not to be made, for it seems to me there is scarcely any symptom in common in the two diseases. This latter is a wholly nervous affection, and while there may be and is at times, great difficulty in breathing, it is attended with no fever; there is no hoarseness of the voice and no croupal cough. In what I have had to say thus far, particularly in that part of this paper which relates to the clinical history and diagnostic features of croup, I have relied mainly on facts obtained by actual observation and personal experience. For in the past few years I have had under medical management at least my share of cases belonging to this disease, and the great fatality attending their treatment has caused me no little mortification and brought up reflections that were painfully unpleasant.

In this connection I would refer briefly to the theory advocated by some, of the *identity* of true croup and diphtheria. This to me seems to be strange conclusion, totally unsupported by clinical facts, and I believe by pathological also. Several years since, while practising medicine in the eastern part of the county, diphtheria prevailed as an epidemic in the neighborhood in which I lived. The affected district embraced an area about two miles wide and three miles long, was elevated and rolling, lying in close proximity to a small running stream, and for years had been comparatively free from sickness of all kinds. The disease broke out about the first of October and continued to prevail until the following February. During its prevalence I treated, alone and jointly, quite a large number of cases. I saw cases representing every phase and character of the disease, from the most malignant down to the mildest grade possible. Except in the very old, no age, sex or condition seemed to be wholly exempt from it. All ages, from the infant of two months to the strong man of fifty years, were numbered among its victims. The vast majority of those attacked however were children probably between two and ten years of age.

Different cases presented different and varied symptoms. In some the first evidence of illness was manifested in a severe and violent chill; this, during the exacerbation of fever, was not unfrequently followed, in children, by convulsions. In others the first symptoms were apparently insignificant and trifling, the patient complaining for two, three or four days of chilliness, headache, slight fever with impaired appetite, but was still able to be up and walk about. But in all cases, however severe or mild, an examination of the throat would reveal more or less redness, swelling and inflammation of the fauces; and if the case had been ailing a day or two there would be found usually on the tonsils, first the characteristic diphtheritic exudation, and if the attack was a mild one the pseudo-membrane would frequently confine itself to these glands. In those of a severe type the entire pharynx up to the velum palati and down as far as the eye could view, would be lined with it; while in cases representing the severest grade of the disease, it invaded the posterior and anterior nares, covered the uvula, soft palate, inside of the cheeks, lips and gums. Exceptionally the larynx and trachea were involved in the exudative process, and of course we then had superadded the usual croupal phenomena. There was in every case swelling of the lymphatic glands of the neck, nearly always, it appeared, proportionate to the intensity of the attack.

In some instances the glandular enlargement was enormous, involving the chain of glands anteriorly, from the base of one ear to the other. The swelling from first to last was characterized by a stony hardness, was generally sensitive to the touch, and in no case was it followed by suppuration. In severe cases there were symptoms not unlike those in mercurial ptyalism ; the saliva, fœtid and acrid, pouring from the mouth in almost a continual stream. From the anterior nares, also, there issued a foul burning discharge, which, in its passage over the lips, would inflame and blister as if by fire. Quickly upon the swollen lips there appear, in many instances, the characteristic exudation. Frequently there was hæmorrhage, not always slight, but sometimes profuse and alarming. The seat of the hæmorrhage was sometimes as extensive as the diphtheritic inflammation, however wide spread that might be. In some of the most malignant cases, there was a peculiar, sickening, nauseous odor of the breath which was almost intolerable. In one or two belonging to this latter class, there was gangrene of the fauces. At one time I picked from the throat of an interesting and intelligent young lady, one among the first cases, both tonsils entire, the whole of the soft palate, with its anterior and posterior arches, and a considerable portion of the contiguous muscular tissue.

Deglutition was always difficult and painful, and sometimes absolutely impossible. It was not uncommon for cases to pass two or three days in succession, totally unable to swallow a teaspoonful of the blandest fluid. A not uncommon symptom was an excruciating pain in one or both ears. Cough was not a prominent symptom, neither in severe or mild cases, excepting in those where there was an involvement of the larynx or trachea, which, as already observed, did not often occur.

The duration of the disease, in fatal cases, varied widely. One bright little girl of four years died in thirty-six hours from the time she first began to complain. In this case the local lesions were comparatively insignificant, death resulting, apparently, from the action of some powerful, general depressant or poison. Some lingered for two or three weeks, and died from asthenia, while there were others still who, after struggling through all the different stages of the disease, with every prospect of an early restoration to health, paralysis of the pharyngeal and palatine muscles supervening, died at last of starvation. Some, after having been convalescing for weeks, were still pale, cachectic and feeble, looking as if they had just passed from the hands of some blighting pestilence—and they had. I just referred to paralysis in connection with the disease. This occurred as a sequel, and, I think, only in the severe cases, and in no instance until after the establishment of convalescence. As to the time of its appearance, and the different parts of the body affected, there was no uniformity or regularity, and in regard to its duration and results, every principle of nervous pathology seemed to be violated.

In some the paralysis was limited to the pharyngeal muscles; in others the palate alone was affected. Some were paralyzed in one arm; another in the leg. One had paraplegia, two hemiplegia. Several had facial paralysis, affecting one side only. Strabismus was not uncommon. One patient had converging strabismus in one eye, and diverging in the other. I think, as a rule, the paralysis was confined to the motor system of nerves, special and general sensibility, apparently, being but little impaired, though this was a point it was difficult, in some cases, to decide. Excepting in one or two quite young children, in whom the pharynx and palate were paralyzed, rendering deglutition impossible, death resulting from inanition, every case of which I had any knowledge recovered perfectly from the paralysis in periods ranging from three to eight weeks. I did not examine the urine for albumen, but have quite recently had under treatment several severe cases of the disease, where I examined urine and found it present in considerable quantity.

I trust I will be pardoned for this seeming digression, and for the time consumed in enumerating some facts I have learned while at the bedside of those suffering with this frightful malady. I have tried to give some of the clinical and diagnostic facts relating to diphtheria, as they actually occurred, in contra-distinction to those of true croup, which some eminent pathologists claim is identical with diphtheria. While I am not prepared to prove the non-identity or duality of the two diseases, I am no less prepared to accept the theory of their oneness or unity.

All who have had any considerable experience in the treatment of true croup, and have watched its progress and its results, will, I think, concur in the conclusion that it is one of the most dangerous diseases of childhood ; indeed, called upon to give a prognosis in any given case, if I should be governed by my own past unhappy experience, it would almost necessarily be unfavorable.

Dr. Ware, of Boston, high authority in this disease, says ninety-five per cent. of the cases of true croup prove fatal. Out of every one hundred attacked, only five recover ! This is, indeed, a fearful mortality, but, notwithstanding this unfavorable showing, I incline to the belief that many cases that die would get well, could judicious treatment be instituted at the *proper time.* The disease is a deceptive one. In the midst of violent and threatening symptoms there is often a rapid change, apparently, for the better. The child becomes quiet, the distressing dyspnœa in a measure subsides, and the little patient, if old enough, may insist on getting on its feet and running about the room. This amelioration in the symptoms is supposed to be due to the detachment of the exudation. Unfortunately, in the vast majority of cases, the improvement is of short duration. After the lapse of a few hours the membrane is reformed, the child grows rapidly worse, and is very soon relieved by death.

We should, therefore, be careful and not deceive ourselves ; nor should we encourage the hopes of friends, when we have reason to know that these hopes are delusive, and are soon doomed to disappointment. To do so is always a cruel wrong, and not unfrequently results in the forfeiture of that confidence and respect which we might otherwise command. A favorable result may be looked for, if, along with an amelioration of the threatening symptoms, the cough should become loose and the expectoration free. In those that recover, convalescence is usually slow and protracted.

In the treatment of this disease, the profession is forced to acknowledge the humiliating truth that they are comparatively powerless. As the presence of the false membrane is the chief source of danger, an important point to decide is, can its formation be prevented ? Is there any method of treatment, however early commenced and faithfully carried out, that will prevent the fibrous exudation ? Does the exudation depend upon the active character and violence of the inflammation ? The history of the disease, together with the results of treatment, as given by competent observers, would certainly give a negative answer to these interrogatories. I think it may be possible, if the case can be seen in the very beginning, by judicious management, to limit the *quantity* of the deposit on any given surface, and, it may be, also the *extent* of the inflamed mucous membrane ; but we are confronted with the fact that, in the majority of cases, the physician is not called soon enough to accomplish ends so important and desirable. As a rule, long before medical aid is solicited the pseudo-membrane is fully formed, and is gradually and certainly moving forward in its work of death. Is there any remedy which, applied topically, will dissolve or remove the exudation ? Not as yet known.

Bearing in mind then, that the false membrane is already present, on which depends the principal danger; that if life is prolonged a few days it will be loosened and thrown off by the suppurative process which is sure to take place, the indications are plain.

Heroic measures, such as blood letting and the administration of powerful sedatives, should not, in my opinion, be resorted to. The patient's strength ought rather be husbanded for the approaching conflict, by appropriate nourishment, and, it may be, the use of alcoholic stimulants, and such therapeutic means adopted and persisted in as may relieve the engorged condition of the inflamed mucous membrane, remove the submucous œdema, promote expectoration and assist in the detachment of the false membrane. In the effort to accomplish at least some of these results, emetics are, perhaps, more largely employed than any other class of remedies.

There is no question, I think, but what, in many cases, they are productive of good, but their use may be most frightfully abused. They undoubtedly play a very important part in controling the nervous element of the disease ; they promote expectoration, and they have something to do, in the later stage of the affection, in the separation and expulsion of the exudation.

This latter happy effect of an emetic was strikingly manifested in a case of mine a few years ago. The patient was a boy about five years of age, of a delicate and feeble constitution, and had been an invalid all his life.

He had been sick with croup four or five days, and the case was as unpromising as it could well be. Calling during one of his severe paroxysms of dyspnœa, I was urged by the friends, if possible, to do something more for the little sufferer. As soon as it was practicable, I poured down the throat of my patient a solution of sulphate of zinc. In a very brief period this was followed by free emesis, during which it appeared as if he must die from suffocation ; but in the midst of his struggles for life, there was ejected from the mouth, with considerable force, quite a large piece of pseudo-membrane. It was very nearly a complete and perfect tube, was about two inches in length, of a dark or leaden color, soft and friable, and its mucous surface was smeared with pus and mucous. The relief to the patient was instantaneous and well-nigh complete. Convalescence commenced at once and the case recovered. From the long list of emetics some choose one drug and some another. The sulphate of alum is a favorite emetic with many. I have used it often, and can testify to its efficiency. Combined with an equal quantity of ipecac (from one to two grams of each), it rarely fails to provoke vomiting. The sulphate of zinc is frequently used ; compound syrup of squill, and even the tartarized antimony, has its advocates. The one, which for several years I have relied on more than any other, is the sulphate of copper. Its chief recommendations are the smallness of the dose, the prompt manner in which it acts, and the almost complete absence of any depressing effect. To a child from three to five years old, may be given 0.07 or 0.15 grams dissolved in water, and repeated, if necessary, in fifteen minutes. Turpeth mineral is also highly extolled by some. I have never used it, and have therefore no experience to give.

Calomel, with many eminent physicians, is a remedy of high repute in this disease. I have but little faith in the correctness of the theory entertained by some, viz: that its employment in croup, by some peculiar effect upon the blood, prevents the formation of the exudations. It is more reasonable to conclude that the good results which some claim for it, are due to the *thinning* effect it may have on the thickened mucous membrane and the removal of the sub-mucous infiltration. I have used it in many cases, in small doses frequently repeated, and in large ones at longer intervals and have tried to watch and study its effects, but cannot say that I am prepared to speak absolutely in its favor. Inasmuch, however, as it has many strong friends and able advocates, it would be well to give it a trial. To a child four or five years of age, I would give as much as 0.15 grams, combined or not, with Dover powder, every two or three hours, continued for two or three days.

Chloride potash is largely used by many, combined with muriate of ammonia, it is a favorite remedy of Dr. J. Lewis Smith. In the treatment of this affection, I consider it an agent of great value, and inasmuch as it is a solvent of fibrin, it meets indications perhaps met by no other drug. While we may not be able to make a topical application of the remedy to the affected surface, it should be remembered that, taken internally, it is eliminated in the secretions and excretions, hence, in this way, it may come in direct contact with every part of the false membrane. I usually make a saturated solution and give it, combined with simple syrup, in teaspoonful doses, for several days in succession if all goes well.

Local treatment is, in many cases, attended with flattering results; indeed, in the management of croup, many rely mainly on this method of treatment. Of the remedies used topically, nitrate of silver has many champions; a solution varying in strength from four to six per cent. may be applied to the pharynx several times in the twenty-four hours, if thought necessary.

Some advise the introduction of the probang into the larynx, a feat which I would not recommend, and one which, I think, is rarely successfully performed. Among other local substances, now largely used by many, may be mentioned the liquor of the subsulphate of iron, bromine, and carbolic acid. With these I

have had no experience. In this connection I would refer specially to the inhalation of steam as a measure of great value. I have employed it in a number of cases, in some of which, at least, I can speak with confidence of its happy effects. A few years since a member of my own family, a boy six years of age, was attacked with croup in its secondary form, The disease occurred in the declining stage of measles. The child had naturally a fair constitution, but his system had been considerably weakened and reduced by the primary disease.

The croupal symptoms soon became marked and threatening, great dyspnœa and a total extinction of the voice rapidly following on the first appearance of the disease. Every feature in the case seemed dark and gloomy, and death appeared imminent and near at hand. Deciding to try the effect of steam, he was taken into a rather small room, and the necessary preparations made as rapidly as possible. These consisted in simply heating several flat rocks and bricks, and placing them in large shallow pans containing a little water. In this way steam was rapidly generated, and very soon I was overjoyed to notice a change for the better. The steaming process was kept up without intermission for at least twenty-four hours, when—the child all this time comparatively comfortable—it was intermitted. In a very brief period the cough became dry and the breathing labored, and the case was evidently again losing ground. The steaming was resumed and continued incessantly for seventy-two hours, when it was gradually withdrawn. From this time there was a slow and steady improvement, which continued until his health was fully restored. It might be proper to add that the aphonia continued for five weeks.

Throughout the treatment the temperature of the room was maintained at 27° to 32° C., and the atmosphere within literally steeped in moisture. As to the external treatment of the neck but little need be said. Some prefer poultices ; some recommend the use of iced water ; while there are others who blister. Poultices and warm water dressings are, in some respects, superior to all other applications, and I generally give them the preference.

One word more and I will close the already too long and imperfect paper. Failing to relieve one patient by therapeutic measures, the question of the propriety of tracheotomy may come up for our decision. As to whether the operation does or does not save life in this disease, the opinions of the medical world are divided. Some eminent practitioners advise and practice it; others condemn it in the strongest terms. If the published statistics in regard to tracheotomy in croup are reliable, certainly many cases operated upon get well, whether *because* of the operation or in *spite* of it may be a question difficult to settle. My own humble opinion is, that the operation, *timely* resorted to, *may*, in rare cases, save life.

Since the days of Bretonneau and Trousseau, it has been practiced quite largely in Great Britain and France, rather sparingly in the United States, though at present the propriety of the operation seems to be growing in favor with the profession of our own country.

